# Supplementary material for: The Effects of Cathepsin B Inhibition in the Face of Diffuse Traumatic Brain Injury and Secondary Intracranial Pressure Elevation
Source: Biomedicines. 2024 Jul 19;12(7):1612. doi: 10.3390/biomedicines12071612 (PMC11274534; doi:10.3390/biomedicines12071612)
Supplement: Supplementary file 1 [file biomedicines-12-01612-s001.zip › Suppl. Table S1.pdf]

Supplemental Table S1. Scoring rubric for the Whisker Nuisance task.

Behavior Criteria rubric	Scores
**Movement**	
Walking around and moving head around	0
Some walking around (possibly when gently prodded) and head movements both toward and away from stimulus	1
Very little head or body movement even when gently prodded	2
**Stance and Body position**	
Body looks round if sitting or long and straight if walking around, looking up, head sway squinting	0
sometimes that the rat hunches or has pinches sides (not frequent, maybe only once)	1
Hunched and/or sides seem pinched in at hips, head low more frequently (could be associated with a side of stimulation; if it is side specific note the side)	2
**Breathing**	
Normal quick breathing (hard to see)	0
Some panting/gasping (very big breaths that are easy to see in body/haunches)	1
Panting/gasping frequently or very robustly (very big breaths that are easy to see in body/haunches)	2
**Whisker Position**	
Whiskers out almost 90 degrees from snout most of the time	0
Whiskers retracted sometimes but out half the time	1
Whiskers retracted during stimulation of that side consistently (if there is a side preference note the rat's anatomical side that evokes the response)	2
**Whisking response/sniffing behavior**	
Whiskers flicker regularly (only visible without stimulus or when switching sides)	0
Whiskers only flicker every now and then (not really linked to active stimulation on that side)	1
Whiskers not flickering at all or not much	2
**Evading stimulation**	
Head and body movements not linked to stick or moving toward stick, Possible sniffing of stick, rearing on hind limbs, head sway (squinting) at stick	0
Movement away from stick a couple times	1

pushing stick with forepaws, clear head or body movement away from stick, hard bite of stick	2
**Response to stick presentation**	
Normal movements either toward stimulus or not linked to stimulus	0
Moving away from stimulus	1
No response (sitting without moving)	2
**Grooming**	
None or normal grooming of less than 30seconds that isn’t linked to stimulus	0
Grooming that lasts more than 30 seconds or is linked to stimulus with possible side preference (occurrences should be clearly divided by non-grooming for at least 10 sec) (if side preference note the side that elicits grooming from rat’s perspective)	2
**Ear Position**	
Ears are up and twitching occasionally (should be able to see a bit into the ear canal)	0
Ears are up with some ears flat or forward (not much twitching)	1
Ears are flat back or far forward (can’t see into the ear canal)	2
**Sniffing**	
Yes	0
Yes, but only 1-2 times	1
No	2
**Fur Ruffling**	
Nice smooth fur	0
Fur is slightly frizzy looking	1
Fur is on end	2
**Urination/Defecation**	
Count how many urine spots (>dime size) and poop pellets are on the pad at the end of the 5min trial.	
no urine stains or poop pellets	0
1-4 urine stains or poop pellets	1
5 and > urine stains or poop pellets	2
