# Supplementary figures and images for: The Effects of Cathepsin B Inhibition in the Face of Diffuse Traumatic Brain Injury and Secondary Intracranial Pressure Elevation
Source: Biomedicines. 2024 Jul 19;12(7):1612. doi: 10.3390/biomedicines12071612 (PMC11274534; doi:10.3390/biomedicines12071612)

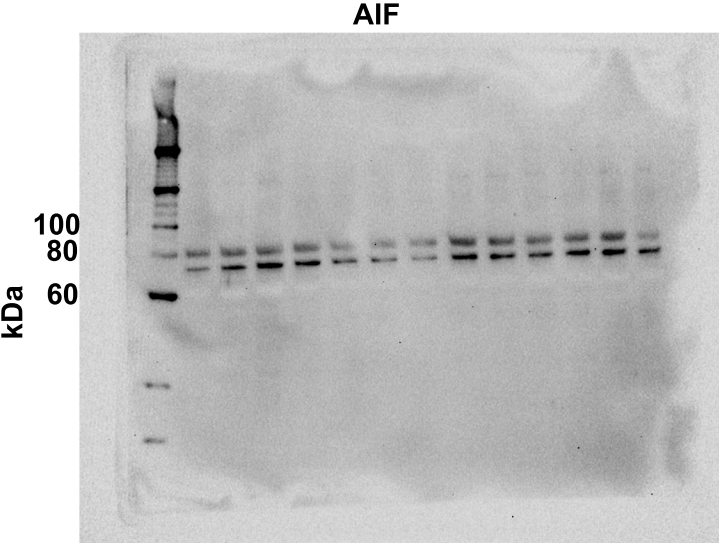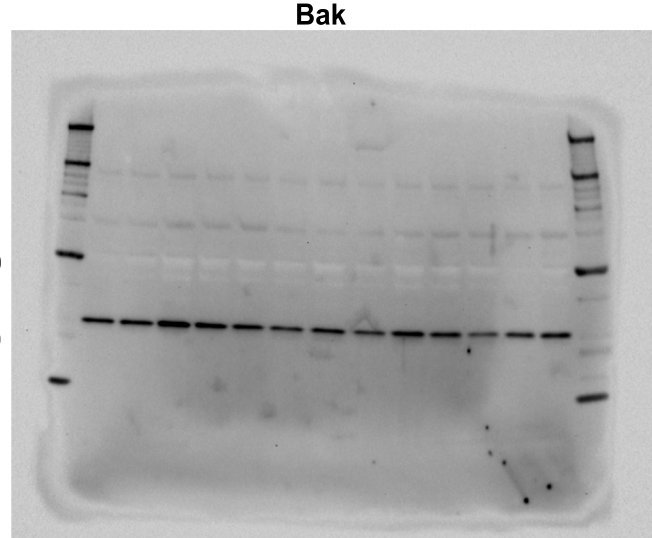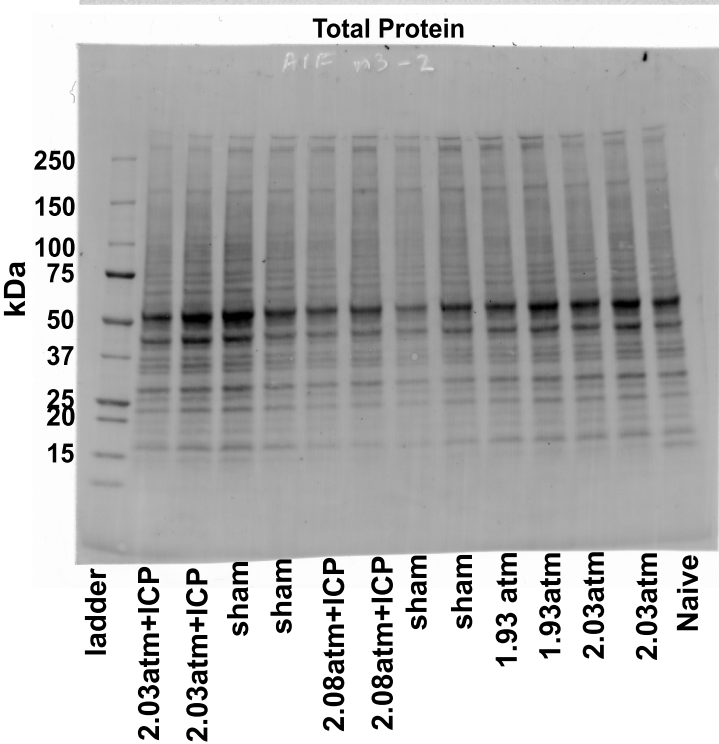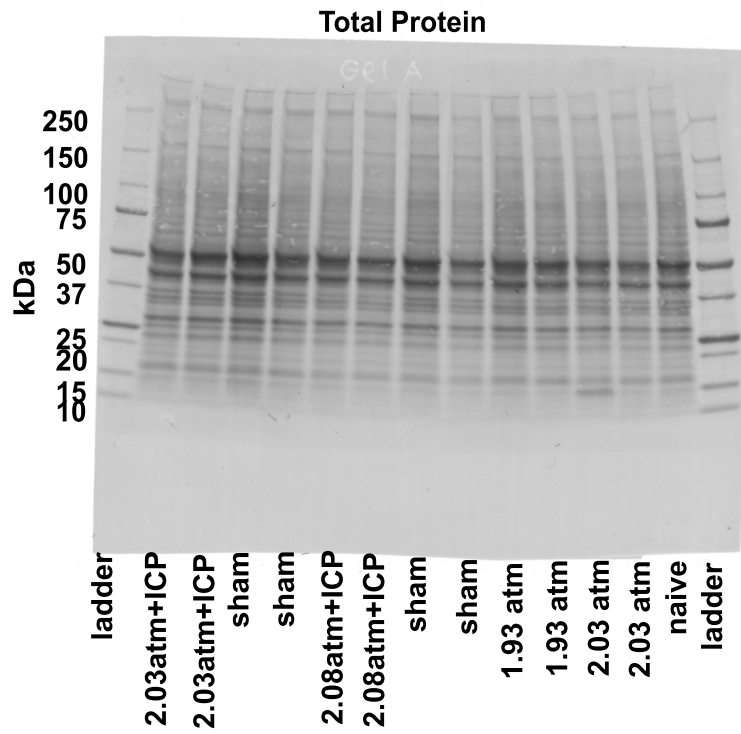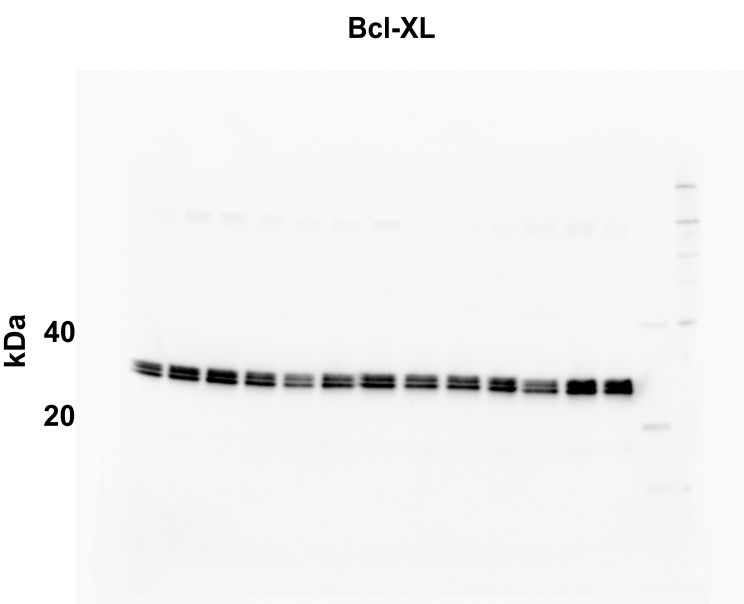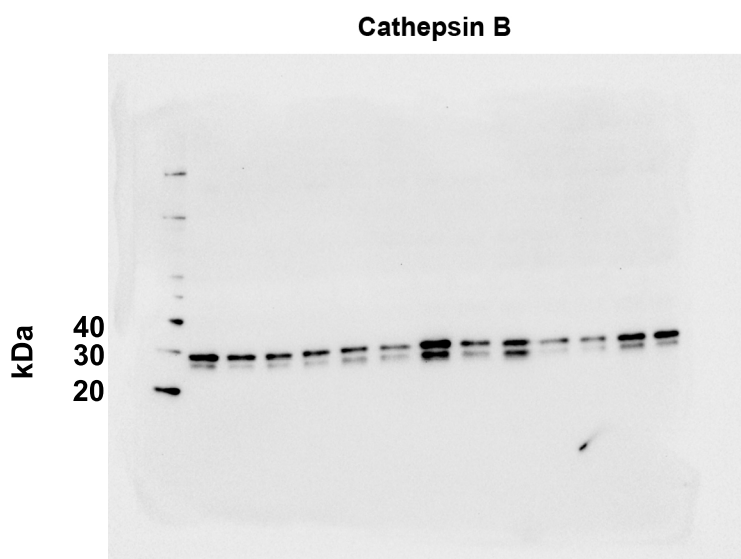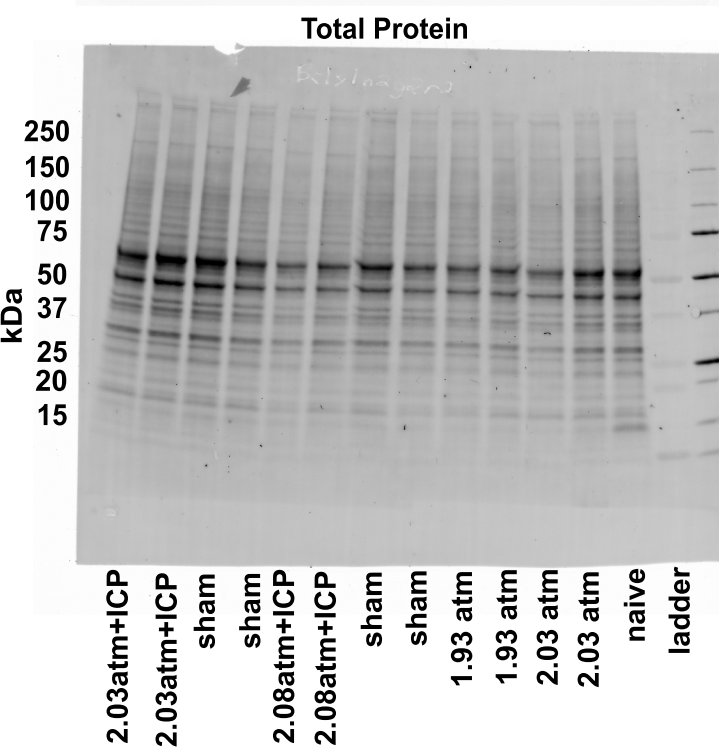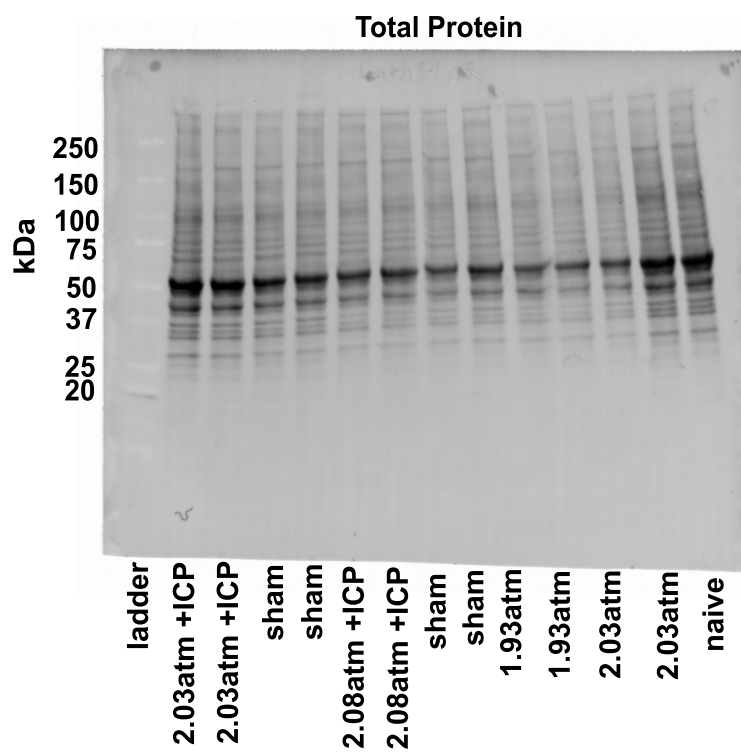

Supplement: Supplementary file 1 [file biomedicines-12-01612-s001.zip › Figure S1. Full Blot Representative Westerns.pdf]
